# ‘I was young, I wanted to return to sport, and re-ruptured my ACL’ – young active female patients’ voices on the experience of sustaining an ACL re-rupture, a qualitative study

**DOI:** 10.1186/s12891-022-05708-9

**Published:** 2022-08-09

**Authors:** Ramana Piussi, Ferid Krupic, David Sundemo, Eleonor Svantesson, Andreas Ivarsson, Urban Johnson, Kristian Samuelsson, Eric Hamrin Senorski

**Affiliations:** 1 SportRehab Sports Medicine Clinic, Gothenburg, Sweden; 2 Sahlgrenska Sports Medicine Center, Sahlgrenska University Hospital, Gothenburg, Sweden; 3 Department of Health and Rehabilitation, Institute of Neuroscience and Physiology, Sahlgrenska Academy, University of Gothenburg, Gothenburg, Sweden; 4 Department of Orthopaedics, Institute of Clinical Sciences, Sahlgrenska University Hospital, Gothenburg, Sweden; 5 Centre of Research On Welfare Health and Sport (CVHI), Halmstad University, Halmstad, Sweden; 6 Department of Sport Science and Physical Education, University of Agder, Kristiansand, Norway

**Keywords:** Second knee injury, Anterior cruciate ligament, Reconstruction, Revision, Re-rupture, Qualitative, Interviews, Mixed method

## Abstract

**Background:**

Despite anterior cruciate ligament (ACL) re-ruptures being common, research on patient experiences after knee trauma has primarily focused on the time after primary ACL reconstruction. Integrating qualitative research and patient experiences can facilitate researchers and clinicians in understanding the burden of an ACL re-rupture. The aim of the study was to explore the experiences of an ACL re-rupture journey in young active females aiming to return to knee-strenuous sports after primary ACL reconstruction.

**Method:**

Fifteen young (19[range 16–23] years old) active females who suffered an ACL re-rupture were interviewed with semi-structured interviews. Qualitative content analysis using deductive approach based on Wiese-Bjornstal’s ‘integrated model of response to sport injury’ was used.

**Results:**

The results are presented in two timelines 1) from first ACL injury to ACL re-rupture, and 2) from ACL re-rupture to present day, and further stratified according to the domains of the ‘integrated model of psychological response to injury’. Results in the first timeline are summarised into seven categories: Finding hope for the journey; Accepting my ACL injury; I succeeded; What matters now? Who am I?; Where will this end? What is going to happen? In the second timeline, eight categories were identified: Fighting spirit; A helping hand; Working hard; I am a new me; I am destroyed; Loneliness; Painful changes; and, I could have made it to the pro´s.

**Conclusion:**

Young active females who suffered an ACL re-rupture did not express any positive experience following their first ACL injury, however, in contrast, expressed positive experiences and personal growth after going through the ACL re-rupture journey, characterized by a lot of struggling, and ultimately led to the experience of becoming a new, stronger person.

## Introduction

An anterior cruciate ligament (ACL) injury is a common sports-related injury, treated with rehabilitation, with the option to also surgically reconstruct the ruptured ligament [1]. During the often long and demanding rehabilitation, patients experience physical and psychological responses as part of their recovery after ACL injury. Patients after ACL reconstruction have been reported to experience uncertainty for full recovery [2], a loss of identity as a consequence of the injury [3], and fear towards having to live through the process of surgery and rehabilitation again [4]. Fear has consistently been reported among patients following an ACL reconstruction [5, 6], inferring that psychological responses, such as fear and uncertainty, perceived as barriers for not resuming pre-injury activity, can be present during rehabilitation following ACL reconstruction [2]. To describe common psychological responses to sport injury, Wiese-Bjornstal’s [7] ‘integrated model of response to sport injury’ is widely used. According to the model, psychological responses of an injury can be cognitive, emotional and behavioural. These responses are dynamic and change over time, with the injured individuals’ personal and situational factors acting as moderators.

After an ACL reconstruction, up to 14% of patients go on to suffer a graft rupture, an ACL re-rupture [8]. Several risk factors for suffering an ACL re-rupture have been identified, including younger age, high activity level, female sex, and the use of allograft [9–11]. More specifically, young active females have been reported with the highest risk of suffering a re-rupture [12].

Despite ACL re-ruptures being common, research on patient experiences after knee trauma has primarily focused on the time after primary ACL reconstruction. An ACL re-rupture, which is a treatment failure, can have physical and psychological harmful consequences for a patient and is associated with inferior knee function compared with after primary ACL reconstruction [13–16]. A deepened understanding of the experiences after an ACL re-rupture can potentially lead to better support for patients who suffer an ACL re-rupture. An approach for research focusing on patients ‘experiences of two subsequent ACL injuries entails the challenge of a dual experience of injury: the period from the ACL reconstruction to the ACL re-rupture, and the following period from an ACL re-rupture to the present day. Integrating qualitative research and patient experiences can facilitate researchers and clinicians in understanding the burden of an ACL re-rupture.

The aim of the study was to explore the experiences of an ACL re-rupture journey in young active females aiming to return to knee-strenuous sports after primary ACL reconstruction.

## Material and methods

### Study design and patients

This study was performed as a qualitative study where data was collected via semi-structured interviews with active female patients who had sustained an ACL re-injury. The first and second ACL injury, and the patients’ experiences of the respective treatment, are unique and highly subjective experiences, built on memories, sensations, feeling, impressions and interaction with others, which motivated the choice of a qualitative study design.

Transcripts from the individual interviews were analysed with qualitative content analysis as presented by Graneheim and Lundman [17]. This method was chosen since our aim was to explore experiences of patients of a certain event and the consequences arising from the event. We used a constructionist theoretical framework, which assumes that reality is constructed through interaction between a subject and a certain event, and therefore, the creation of meaning requires a mind or person, and a social context. Consequently, the concept of ‘reality’ is dependent on the individuals interpreting it (relativist ontological position) [18]. As an epistemological standpoint, we have a subjectivist/transactional position, inferring that knowledge is co-constructed through interaction between researcher and data [19]. The Consolidated criteria for Reporting Qualitative research (COREQ) [20] checklist was used to report transparent methodological information.

An interview guide (Table 1) was developed by the first (RP), the third (DS), the fourth (ES) and the senior author (EHS) through extensive discussions and screening of the literature on the subject. A draft of the questions was then sent for confirmation to the second author (FK) with experience in qualitative research. This process was repeated until consensus was reached among all the authors.

**Table 1:** Interview guide

*Sports and leisure time*
Do you perform any sport as today?
What does your sport mean for you?
What are your dreams with your sport participation?
What do you do besides sport (work/study)?
Has your injury influences your choices of work?
*First ACL injury/rehabilitation*
How did you injure your ACL the first time?
How was the rehabilitation after the first surgery?
Do you recall anything particularly tough during rehabilitation?
Do you recall anything particularly easy during rehabilitation?
Was there anything that made you particularly sad during rehabilitation?
Was there anything that made you particularly happy during rehabilitation?
Did you get the support you wished during rehabilitation, and if so, from whom?
*Second ACL injury/rehabilitation*
Can describe how you injured your ACL the second time?
How did you feel?
Did you directly know it was the ACL again?
How did you feel upon receiving the medical notification that your ACL was ruptured again?
Did you feel anything was missed during your treatment – if so, what?
What did you feel about having to go through another rehabilitation process?
How do you think rehabilitation has affected you as a person?
How do you think suffering two ACL injuries as affected you as a person?
How did you look at the future before the second ACL injury?
How did the second ACL injury affect the way in which you looked at the future?
How did the second ACL injury affect your ambitions?
*Social life/personality*
How do you think your ACL injuries have affected your social life?
What kind of support have you had along the way?
What did your teammates mean to you?
Have you been spending less time with your teammates because of the ACL injuries? If so, why?
Do you feel lonely? If so, why?
How has the ACL injuries affected your self-image?
Is there anything related to your knee that you are afraid of?
If you could go back in time, what would you change?
How do you feel today?
Do you have any proposition for improvement?
Is there anything you would like to add?

The patients were recruited through verbal advertisement by sports physiotherapists at different rehabilitation clinics in Sweden. Patients were then contacted by telephone by first author (RP) to confirm that patient were eligible for inclusion: female sex, age between 16–25 years, at least level 6 on the Tegner activity scale before their ACL injury, i.e. previous regular participation in knee-strenuous sport (defining “active”), and the occurrence of an ACL re-rupture. Patients were informed about the study and asked whether they were interested in participating. Upon positive response, an interview was scheduled. No patient declined participation, and the final cohort consisted of 15 patients (Table 2). The choice to include 15 patients was taken at it was deemed realistic to reach data saturation, which was assessed during analysis. All the patients were informed that participation was voluntary and that withdrawal from participation was possible without any explanation at any time. All the statements from patients were analysed confidentially. Written consent was collected. Ethical approval has been obtained from the Swedish Ethical Review Authority (registration number: 2020–02834).

All interviews were carried out by the first author of the study. Patient demographics are presented in Table 2.

**Table 2:** Demographics of patients

*Patients (n* = *15)*	Mean (range)
*Age*	19.1 (16–23)
*Height (cm)*	169 (162–184)
*Weight (kg)*	63.3 (58–79)
*BMI*	22 (19.1–23.7)
*Time between index reconstruction to re-rupture (months)*	13.4 (4–26)

*Cm Centimetres, kg Kilograms*

During the development of the interview guide, our bias was that this specific group of female individuals who had sustained a ACL re-injury could possibly suffer from symptoms of anxiety, depression, have low athletic identity, and perceive a relatively low health status. Because of this we asked patients to complete the following patient-reported outcomes (PROs) at time of interview: the Short Form (36) Health Survey (SF-36); the Hospital Anxiety and Depression Scale (HADS); the Montgomery Åsberg Depression Rating Scale (MADRS), and the Athletic Identity Measurement Scale (AIMS) (Table 3). Results from a systematic review suggests that depressive symptoms are more common in individuals with ACL injury compared with symptoms of anxiety [21]. Therefore, the choice to assess symptoms of depression with two different PROs and symptoms of anxiety with one PRO. Results from PROs were used for demographic purposes only, that is, the results were not taken into account in the analysis process.

**Table 3:** Patient Reported Outcomes used in the present study

*PRO*	*Aim*	*Questions*	*Score*
*Short Form Health Survey (SF-36) *[22, 23]	To survey health status in medical studies	36 items divided into eight domains of health status	From 0 to 100, with higher scores indicate better health status. Scores calculated with an on-line calculator
*Hospital Anxiety and Depression Scale (HADS) *[24, 25]	To assess the magnitude of symptoms of anxiety and depression	16 items divided into 2 subscales, scored 0–3	21 points being the highest score for each subscale, representing the highest severity of symptoms, with a value ≥ 8 representing depression or anxiety [25]
*Montgomery Åsberg Depression Rating Scale (MADRS) *[26]	To assess severity of depression in depressed patients	10 items, scored 0–6	Answers are summed to a total score, with 0 indicating no depression and 60 indicating extremely severe depression
*Athletic Identity Measurement Scale (AIMS) *[27]	To measure athletic identity (i.e. the self-identity of athletes in relation to the sport domain)	10 items, scored 0–5	Higher scores (50 being highest) on the AIMS reflect a stronger investment in being an athlete as a source of self-worth

In order to improve transparency and reflexivity with qualitative research, the first and the senior author are experienced physiotherapists working in a sport rehabilitation setting (years of experience, 5–10), with a Master of Science (MSc) (first author) and a PhD (associate professor, senior author). Preconceptions about the subjects were extensively discussed between the first (RP) and the senior (EHS) author throughout the analysis process. In terms of the other co-authors, one (FK) works as a registered nurse (PhD), three are medical doctors (KS, professor; DS, PhD; ES, PhD), all having experience of qualitative research, and two authors (UJ; AI) are psychologists, with PhDs in psychology (both professors) with extensive experience in the sports psychology research field. All authors except one (ES) are males. The senior author (EHS) was the responsible physiotherapists for one of the patients at the time of the study. No other direct relationships were present between any of the patients participating and the authors of the study.

### Data collection

Data was collected between September and November 2020 in Gothenburg. Due to the COVID-19 pandemic, not all interviews were performed in a physical setting; nine interviews took place in a sports rehabilitation clinics conference room, and six interviews were performed digitally (via Zoom™, web-based application (Zoom Video Communications, Inc. San José, California, USA)). No other person beside the interviewer (RP) and the patients were present at the time of the interview. During the interviews no field notes were taken, and the first author provided neither own bias nor assumptions, in order not to bias the informants. Interviews were recorded with a laptop (physical interviews), or via the Zoom recording function (electronic interviews). Interviews lasted between 21 and 47 min, and records were transcribed verbatim, resulting in 112 pages. Transcripts were not sent to participants for corrections or comments since validity implication of this methods are questionable [28], and to minimize patient burden.

### Data analysis

**Fig. 1:**
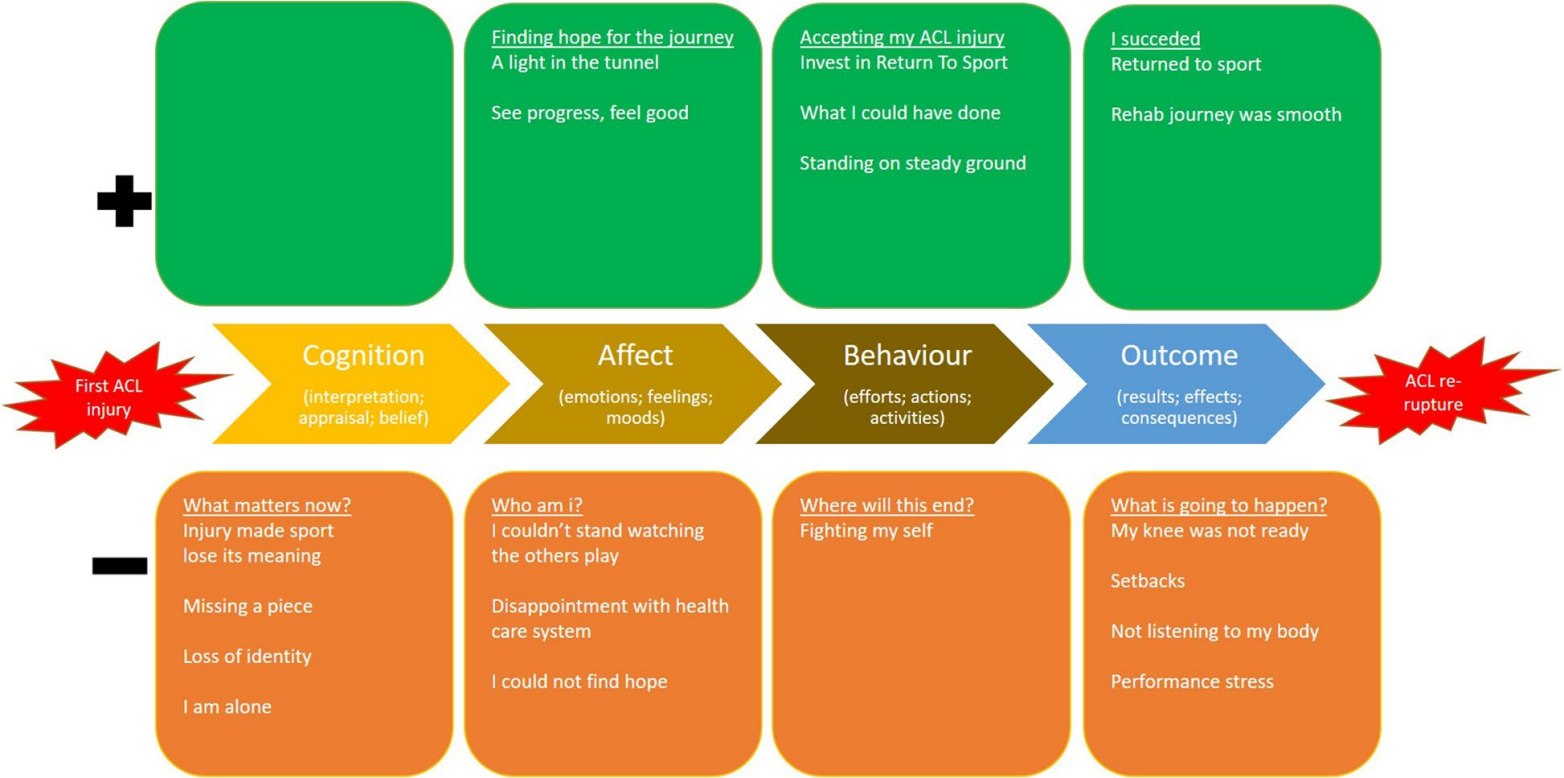
Main and sub-categories on a timeline with the ACL injury as a baseline and ACL re-rupture as an endpoint. ACL: Anterior Cruciate Ligament

**Fig. 2:**
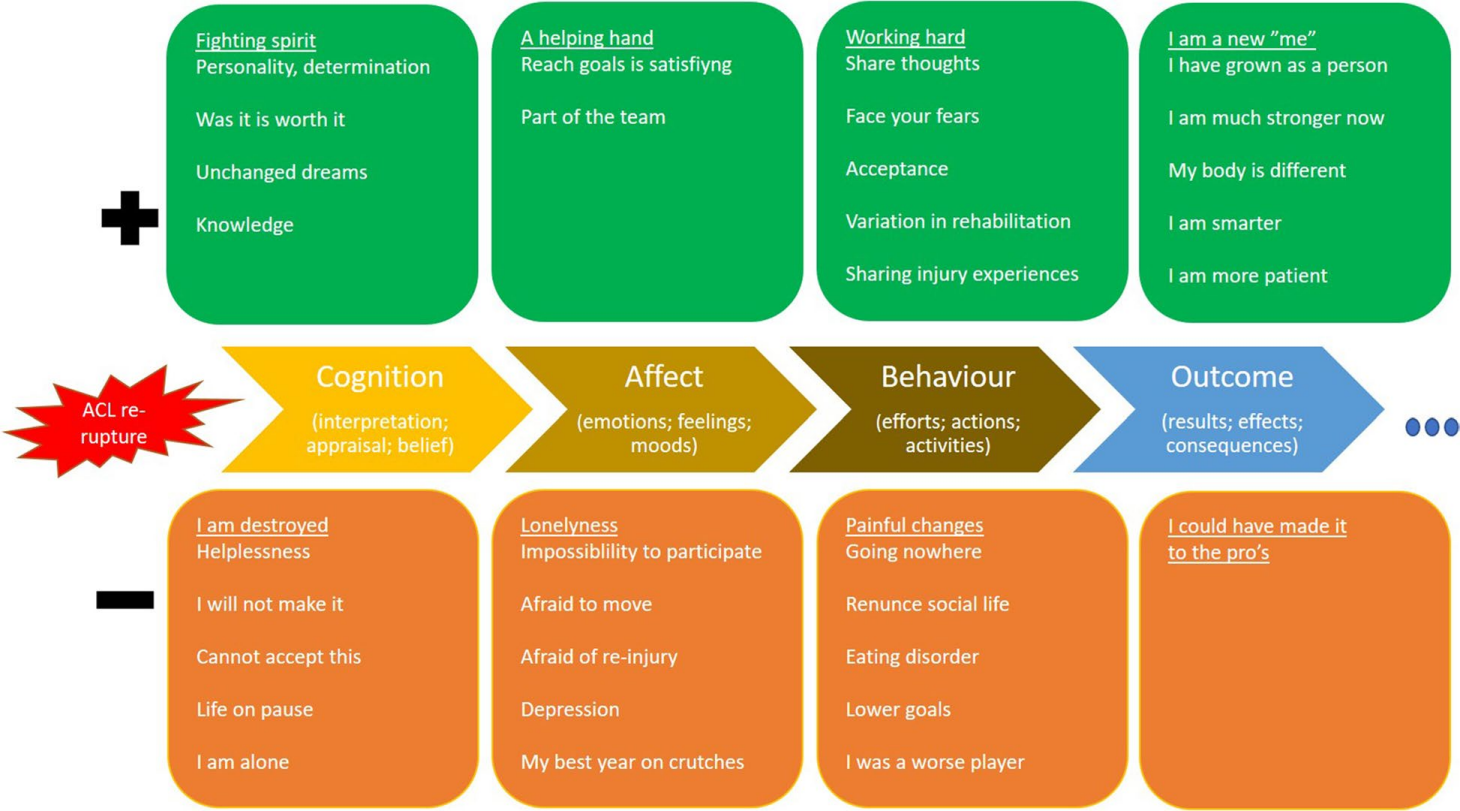
Main and sub-categories on a time line with the ACL re-rupture as a baseline and no endpoint. ACL: Anterior Cruciate Ligament

The data were analysed using qualitative content analysis based on Graneheim and Lundman [17, 29]. The first (RP), the fifth (AI), the sixth (UJ) and senior (EHS) authors were responsible for the data analysis process. Transcripts were read thoroughly to create a general understanding among authors. Secondly, meaningful units were extracted and shortened into condensed meaningful units. The condensed meaningful units were then abstracted and coded. To address trustworthiness, during the second step, data was triangulated among the research team, where authors in the research team were invited in the analysis and asked for feedback in the identified main- and subcategories. Codes addressing similar categories were grouped into sub-categories, and sub-categories were then grouped into main categories. Upon completion of data analysis, a judgement was made that data saturation was achieved. During this process, we chose to use a deductive approach based on *the "integrated model of response to sport injury"*. [7] We acknowledge that Wiese-Bjornstal´s [7] model is presented in a predominantly shape of a circle, and states that psychological response to an injury is cyclic, and components are inter-related. However, the model assumes that cognitive appraisals affect emotions, which in turn affect behaviours. Because the aim was to explore the experiences of a journey leading to an ACL re-rupture in a population of young females, the results were summarised in a timeline, starting from the first ACL injury, through rehabilitation, to the second ACL injury, and through rehabilitation again. Therefore, we chose to present results on two lines comprising the four domains from in the model to create a visual representation of our results (Figs. 1 and 2). We grouped sub- and main categories into the different domains of the model, stratifying categories into positive or negative responses. During the process of abstraction, coding and categorization of codes, the interview transcripts were repeatedly read to ensure that data was appropriately understood in relation to the context, and therefore ensure credibility. Any disagreement between authors was resolved by discussion with the senior author (EHS). Credibility is a crucial part of trustworthiness in qualitative research, [29] therefore, after grouping categories into sub-categories, the transcripts were read again and sub-categories were validated against the transcripts, in order to ensure that data were not missed or erroneously included.

## Results

The mean time between primary ACL reconstruction and ACL re-rupture was 13.4 months (range 4–26 months) for the 15 included young active females (Table 4). At the time of first ACL injury, 5 young females were active in football, 3 in handball, gymnastic, and floorball, respectively, and 1 in basketball.

**Table 4:** Months from index ACL reconstruction to return to training with restrictions, return to unrestricted sport, and ACL re-rupture for each patient

*Time in months from index ACL reconstruction to:*					
*Patient*	*Return to training with restrictions*	*Return to unrestricted sport*	*ACL re-rupture*	*Time of interview*	*Treated with ACL revision*
*1*	9	10	10	29	✓
*2*	5	15	23	42	✓
*3*	n/a	n/a	4	23	✓
*4*	9	10	10	31	✓
*5*	4	n/a	15	45	✓
*6*	9	12	12	47	✓
*7*	6	10	16	54	✓
*8*	5	10	12	54	✓
*9*	10	12	12	59	✓
*10*	11	12	12	59	✓
*11*	10	12	17	68	✓
*12*	5	11	11	47	✓
*13*	12	24	26	70	✓
*14*	10	11	15	27	✓
*15*	9	11	11	29	✓

*n/a not applicable (patient not reaching stated goal), ACL Anterior Cruciate Ligament, revision: second ACL reconstruction performed after re-rupture*

Descriptively, after having suffered an ACL re-rupture, i.e. at the time of interview, patients in our cohort did not experience symptoms of depression or anxiety, measured with MADRS or HADS, perceived a good level of general health (SF-36 = 85.7), and experienced, on general, medium levels of athletic identity after the two injuries (AIMS = 34/50) (Table 5).

**Table 5:** Answers to included patient reported outcomes

*MADRS*											
Median (range)						5 (0–14)					
*AIMS*											
Median (range)						34 (16–48)					
*HADS*											
		*Depression*						*Anxiety*			
Median (range)		1 (0–4)						6 (1–9)			
*SF-36*											
	*Physical functioning*		*Role physical*	*Role emotions*	*Energy/ vitality*		*Emotional well being*		*Social functioning*	*Pain*	*General health*
Mean (range)	87.3 (75–100)		76.7 (0–100)	84.5 (33–100)	68.0 (40–80)		79.7 (60–96)		89.2 (63–100)	81.5 (58–100)	85.7 (70–95)

*AIMS Athletic Identity Measurement Scale, highest score 50 (strongest athletic identity), HADS Hospital Anxiety and Depression Scale, highest score 21 (most severe symptoms) for each subscale, MADRS Montgomery Åsberg Depression Rating Scale, highest score 54 (most severe symptoms)*

*SF-36 Short Form Health Survey, highest score 100 (no symptoms) for each subscale: physical functioning; physical role limitations; emotional role limitations; energy/vitality; mental health; social functioning; bodily pain, and general health perceptions*

Main and subcategories (Table 6) extracted from the qualitative content analysis using a deductive approach based on Wiese-Bjornstal’s model [7] are presented in Figs. 1 and 2. Figure 1 presents the journey between first ACL injury and ACL re-rupture, while Fig. 2 presents the journey from ACL re-rupture to where patients were at the time of this study.

**Table 6:** Main categories stratified as positive or negative in the journey from ACL injury to ACL re-rupture, and from ACL re-rupture to the time of interview, respectively

**ACL injury to ACL re-rupture**				
Positive		Finding hope for the journey	Accepting my ACL injury	I succeeded
Negative	What matters now?	Who am I?	Where will this end?	What is going to happen?
**ACL re-rupture to present day**				
Positive	Fighting spirit	A helping hand	Working hard	I am a new me
Negative	I am destroyed	Loneliness	Painful changes	I could have made it to the pro´s

*ACL Anterior Cruciate Ligament*

### The journey from ACL injury to ACL re-rupture

#### Cognition

During the journey from ACL injury to ACL re-rupture, patients experienced feelings of loss on multiple levels. Patients experienced that the ACL injury led to their sport losing its meaning. Furthermore, patients experienced losing a piece of their own self as they were forced to disrupt sport participation because of the ACL injury. Not being able to participate in their sport that characterised the patients’ life before ACL injury, made patients feel that they could not recognise who they were anymore, leading to a loss of identity, in which patients especially experienced loneliness. Experiencing feelings of loneliness and loss, resulted in a main category *‘what matters now?’.*

*‘[…]it is so sick that something (the sport) … can mean so much. It is like a second home. It really is. When you grow up with the sport and the happiness that the sport gives you and…. To disappear from it... It has been extremely hard’.*

No positive experiences were identified after the first suffered ACL injury in the analysis.

#### Affect

During the journey between ACL injury and ACL re-rupture, experiencing sense of loss in the cognitive level, resulted in desperation, depressive responses, and in that patients felt they could not find hope to move forward in life: it was hard for patients to see how the situation could get better. Patients experienced that it was unbearable to go to the team´s training and see all teammates training, and not being able to participate. Furthermore, some patients went through a difficult period of time before they received their diagnosis of ACL injury. A difficult diagnosis process made patients experience that the healthcare system could not support them properly and they had to seek help at different medical caregivers before receiving any help. Because of this, patients felt disappointed towards the healthcare system. These negative affective responses contributed to patients experiencing uncertainty and resulted in a category of *‘who am I?’.*

*‘[…] it is like they put you aside… when you are injured… like… a ‘B’ group when you are injured. It is like you do not belong anymore’.*

*‘[…] if I could change something, I would directly go to her (the ‘new’ surgeon) instead for him (the first surgeon). He was surely good, but he did not listen to me. I said it was wrong… I said I was in pain. Something was wrong in my knee… but he was like… no everything feels fine with the knee… but if I went directly to her who operated me the second time… maybe I could have… been playing again…’.*

On the other hand, patients experienced a strong desire to return to sport, and to leave the ACL injury behind, which was seen as a ‘light in the tunnel’. During the rehabilitation period, patients experienced seeing progress (getting stronger, faster, and perceiving less pain) as satisfactory. In the shades of an ACL injury, these aspects made patients *‘finding hope in the journey’.*

*‘[…] immediately after surgery (first ACL reconstruction) it was the hardest period. The period following the first moments after surgery was better, since you come into the routine, you go to rehabilitation and train. Then you start seeing small improvements… and… those improvements trigger you to keep on going and fighting. And then, when some time has passed, you get to start with tougher exercises, and then running… and it keeps on triggering you… to fight even more… or at least that´s what happened to me’.*

#### Behaviour

Patients experienced that something was wrong with their body, but they did not get the help, support or necessary attention to properly take care of the ACL injury. Such experiences of expectations for help and support not being met made patients feel they were fighting their own self. Patient felt that something was wrong but at the same time were not being listened to, and no matter how patients would behave, the future was uncertain, which led to a main category of *‘where will this end?’.*

*‘[…] I think it is important to speak about this. I have had a journey not many have had. This ‘being sent’ forth and back between different caregivers... I have felt all the time that something was wrong, and every single time I was answered that nothing was wrong, that possibly my kneecap was not totally straight… and that I should just keep fighting, like I was not fighting enough! And then I met the other surgeon and he keeps asking if I am certain that I have problem with my kneecap… No I don´t know! This is what I was told… I have no idea… How can I know what is the problem? I just feel my knee is wrong. And then (after MRI) they tell me that my ACL is floating around like seaweed, and because of that my meniscus is now damaged. And I thought I was ready to return to play…’*.

At the same time, seeking for help, and receiving proper support by important individuals in the social context, and by teammates, made patients experience a steady ground to stand on. From the steady ground, it was possible to make the necessary investments in time, energy and effort to aim for returning to sport. Thinking retrospectively about their journey, patients wished they put more energy into strength training and rehabilitation. A key concept in the primary part of the journey was *‘accepting the ACL injury’* in order to gather the energy and motivation to carry on with the rehabilitation.

*‘[…] I had amazing support from my physiotherapists… We were close. We talked a lot and I had all the chances to ask all the questions that were running around in my head… and we had mental support from the club as well…so even if you are alone in that situation, meaning that you alone have to do the work, and you have the injury… but still… and I had support from my family and from other people around me… it meant a lot for me’.*

#### Outcome

When approaching the time to RTS after their first ACL reconstruction, patients experienced that their knee was not ready, but they felt forced to show up and perform at trainings, even though they did not feel ready for the sporting situation. Patients experienced several different set-backs on physical (pain, swollen knee) and psychological (loss of motivation, questioning the meaning with sports) domains, which led patients to feel they were not listening to the body, and therefore wondering *‘what is going to happen?’,* while experiencing performance stress.

‘*[…] that weekend we were supposed to play a game, so I asked the coach to train on Thursday as well. We just had to practice as much as possible before the game. That Thursday I had my ACL re-rupture. We already had played a game on Wednesday so my body was tired…. But I…. my motivation was…. We had to be better. We were good enough on Wednesday to meet the opponent team the following weekend*’.

However, for some patients, the rehabilitation from ACL injury to RTS was a smooth journey, and upon returning to sport, patient experienced feeling of completeness and success, resulting into a main category of ‘*I succeeded*’.

‘*[…] at that time (upon return to sport) … everything went so good. Rehabilitation… and I was… everything fell in the right place when I was back (returned to sport). It felt great. I was so positive. No doubt it would just work. I just thought… now it goes straight forward. No way it (the ACL re-rupture) could ever happen’.*

Unfortunately, for all patients, the final outcome of the first part of the journey was an ACL re-rupture.

### The journey from ACL re-rupture to the present day

#### Cognition

After suffering an ACL re-rupture, patients experienced that it was impossible to accept what happened. Patients experienced feelings of loneliness on a deeper level compared with after the first ACL injury, and experienced that life could not continue, therefore, patients felt life was put ‘on hold’. Furthermore, patients felt helpless as everything they did had not helped them to avoid a new ACL injury. Patient experienced they would not make it through rehabilitation towards a normal functioning body again, therefore patients experienced ‘*I am destroyed’*.

‘*[…] I am 19 years old. It cannot happen. I did everything right. I basically live at the gym. I do everything for my sport… and then it happens (the ACL re-rupture). Why me?... I was crushed*’.

After already having injured and rehabilitated an ACL injury once, patients learned what was required to progress in rehabilitation, and could preponderate whether the long rehabilitation process was worth returning to sport. Upon considering this, patients experienced that their dreams had not changed, and thanks to personality traits such as determination, patients decided to fight their way back, leading to a main category of ‘*Fighting Spirit*’.

‘*[…] it was so hard (getting through the rehabilitation journey) but... I always had the feeling that I am the one who decides if I am going to play or not. There is only one way, and that is forward. It is tough since you know what you have ahead of you (rehabilitation), but you know it is worth it when you get there*’.

#### Affect

Patients experienced being affected by depressive symptoms following the ACL re-rupture. Furthermore, patients experienced fear of re-injury and fear of movement. Patients experienced they could not participate in sport, and in daily activities (e.g. swimming in the sea) with individuals who mattered for them. Not being able to participate was a major psychological obstacle to find motivation. Patients felt that they spent their best years on crutches, which was summarised as a main category of ‘*loneliness*’.

‘*[…] I have several friends who have ruptured their ACL, but it is a huge difference to do it once or twice… so nobody can actually step into my shoes. I can speak with my friends or family, but nobody can really help me completely…*’.

Upon continuing to fight for a RTS, the patients’ positive experiences were summarised in a main category of ‘*a helping hand*’, as patients experienced support from the team they were playing sport with, and being part of the team as a significant factor for finding the strength to keep motivation. Furthermore, patients experienced reaching goals with rehabilitation (reaching goals set on improvement of physical performance) as satisfying.

*‘[…] it was like… when you reached a goal (physical goal set with rehabilitation), you got so happy and felt successful. And even if sometimes it was hard (the rehabilitation journey), I always had support of people around me and I would like to say that it is easier when you feel supported…. And when you reach your goals… it is pure joy and everything becomes possible’.*

#### Behaviour

During rehabilitation, patients experienced several ‘*painful challenges*’. Patients felt that rehabilitation was not progressing, and they were not reaching pre-determined goals, therefore feeling that they were going nowhere, no matter the effort patients would put in training. Furthermore, patients experienced they could not perform as well as before the injury, and felt they could not contribute to the team as they did before. The difficulties in participating in social activities mentioned previously, led to patients renouncing social life and giving up on friends. In some patients, the experienced symptoms of depression led to eating disorders and compulsory training.

*‘[…] I got so sad, I started feeling so bad so I developed an eating disorder. I could not train as I did before. […] and then training became instead destructive. I trained to punish myself’*.

However, patients felt that seeking other patients in the same situation to share thoughts and sharing ACL injury experiences was a great remedy for not feeling as lonely. Having a physiotherapist listen to patients and create variation in rehabilitation with different exercises and different training set-ups was appreciated by patients during the rehabilitation. Furthermore, patients experienced that facing their own fears and accepting fears and the situation they were in led to significant positive progression in the journey towards, resulting in a main category of ‘*working hard*’.

*‘[…] I had many people around me that pushed me and kept on saying I could succeed… it was important. But… I think it is more about… I decided that … I mean, I love the sport, and this is what I want to do… and… now I am in this situation, but I did it once already. Why wouldn´t I be able to do it again? I decided to give it everything I had. But… I was too isolated in my own bubble in order to make the effort… ‘[…] my fear (of ACL re-injury) is not an obstacle. It is there, but it is something to overcome rather than something that stops me.*

#### Outcome

Several patients included in this study had not returned to their preinjury sport level after an ACL re-rupture. One main category summarising negative thoughts and regrets was that patients felt *‘I could have made it to the pro’s*’ if they had not suffered an ACL re-injury.

‘*[…] if everything was done right from the beginning maybe I would still play and had become an elite player. But everything that happen (setbacks) made me give up hope to come back’*.

However, as positive outcomes from going through an ACL re-rupture, patients felt they grew as individuals, and patients experienced a new inner strength with stronger determination and resolution. Patients felt they became smarter than before their ACL re-rupture journey, and that they have become more aware of their body, which they perceive differently, and that they listen to their body. Furthermore, patients expressed being calmer and to have more patience compared with before the ACL re-rupture journey. Finally, patients felt that the ACL injury process and having sport forcefully taken away from them, led to more leisure time to cultivate their own interests, meeting new friends outside the sporting activity, and focusing on different parts of life, resulting in a main category of ‘I am a new me’.

*‘[…] I think it has changed me a lot. I do not think I would want to be without (the ACL injury process). Or, I mean… Maybe I would want to be without (the ACL injury process) since I could play (the sport). But I have a different mental strength now. I can help people in a different way. I have become more supportive and I understand other people differently. And then, manage to pull yourself through two big things (ACL injuries) will… I am young now but … in the future… I have learned how to handle things and think positive. It is extremely hard (the ACL injury process) but it has shaped me as a human being and made me to somebody who does not give up.’*

## Discussion

The main results of this study were that young active females who go on to suffer an ACL re-rupture summarised the first ACL injury as a negative incident, however in contrast, expressed positive experiences and personal growth, reflected by the feeling of ‘a new, stronger, me’ after going through the ACL re-rupture journey.

The main categories resulting from this qualitative content analysis were categorised into positive and negative subheadings. Despite the *integrated model of respone to sport injury* [7] being presented as a circle, we chose to present the results on two timelines, starting from 1) ACL injury to the ACL re-rupture, and, 2) from the ACL re-rupture to the time of interview. We deemed the choice to adapt the psychological response to a timeline as appropriate for our data based on our clinical experience. However, we acknowledge that the psychological responses experienced by the patients are not one dimensional, and main categories resulted from data analysis are likely occurring at the same time, and interchanging with each other throughout the journey.

The results of our study are novel in that we interviewed young active female patients who have suffered an ACL re-rupture, giving them the unique opportunity to explain factors they believe important for their ACL journey with their own words. The results suggest that patients were deemed ready to RTS by healthcare professionals, however, not feeling ready to return. Despite previous studies reporting impaired knee-related quality of life in ACL deficient individuals, [30] and ACL reconstructed individuals at least 5 years after surgery, [31] the patients in our study reported positive experiences, feeling as a new, stronger person, and reported that their knee injuries allowed them to have the opportunity to explore other interests in life. Therefore, to evaluate knee-related quality of life alone, may be insufficient for a holistic and long-term reflection of patients’ satisfaction with the treatment after ACL injury. In addition, symptoms of depression have been reported in patients 5–20 years after ACL reconstruction, [32] but in our study, we did not identify symptoms of depression or impaired self-reported health in the descriptive responses from the MADRS, HADS, or SF-36.

When patients were close to returning to sport after their index ACL reconstruction, setbacks were experienced, including pain and swelling of the knee joint, and patients felt they could not completely trust their knee. Patients expressed that their knee did not feel ready for returning to sport, despite passing RTS criteria in a clinical test battery. Therefore, based on the experiences of the patients in our study, the clinical tests we use to assess whether patients are ready to RTS after an ACL reconstruction, do not necessarily comply with whether patients feel ready to RTS. The difference between passing clinical tests and patients’ subjective feelings has recently been highlighted in previous publications, as patients state fear of re-injury as a main component for not returning to sport, [33]. At the same time, RTS criteria seldom comprise the assessment of fear, and may not identify a subset of patients at risk of sustaining an ACL re-rupture [34, 35]. This mismatch between how clinical healthcare professionals interpret results from clinical tests, and how patients feel about returning to sport, is challenging, especially with respect for shared decision-making. In this aspect, we argue that that the patients’ perspective in shared decision-making for RTS needs further emphasis, and, structured methods to ensure its inclusion is warranted [36]. Although current RTS testing is likely beneficial to determine the minimal level for clearance of returning to sport participation, it must be acknowledged that some of the current objective RTS criteria are of arguable validity and do not capture how patients feel about RTS. We encourage the use of clear and transparent sharing of information from the responsible healthcare professionals, while the final decision of RTS should be taken by the patients themselves.

The patients in this study expressed that early and limited participation in their sport after ACL reconstruction could be detesting, where patients felt that they only could watch teammates play the sport, while they were standing on the side line. It has previously been reported that patients believe social support is crucial for a successful rehabilitation [37]. However, the interpretation that social support is passive and enforced by allowing patients to perform rehabilitation tasks at the same time and place where other teammates train, may have been made by healthcare professionals, and never been validated by patients themselves. Some of the patients in the present study mentioned that being part of the team and being able to participate in team activities, such as trainings, was important for returning to sport and finding the strength and motivation to proceed through rehabilitation, a finding which is in accordance with previous research on elite female football players [38]. Whether social support should be provided ‘on field’ or not is likely individual and should be adapted from patient to patient.

Experiences of women suffering a second ACL injury have been previously described, where women expressed that the rehabilitation after the second ACL injury as a lifelong adaptive coping process sometimes without the possibility of returning to previous activity levels[39]. There are similarities in patient’s experiences in our results and results presented by Heijne et al. [39], where patients experienced the first injury as more difficult to cope with compared with the second ACL injury, due to lack of knowledge and difficulties in setting expectations, and all patients experienced the rehabilitation as time-consuming. On the other hand, there were differences between the studies, where patients in Heijne et al. [39] changed their goals for rehabilitation between the first or the second ACL injury, where returning to sport was changed to returning to a normal life. In our study, several of the included patients were treated with an ACL revision and returned to pre-injury level of sport. Furthermore, our main results where patients expressed personal growth and strength as an outcome of the injury process were not reported by Heijne et al. [39]. These differences can be related to the patients in our study being substantially younger (16–23 years compared with 17–36 years). 

### Methodological discussion

Since we moved from a constructionist theoretical framework, which assumes that reality is constructed through interaction in human practices between a subject and a certain event, it is possible to assume that different patients have different experiences of the same event. Therefore, since qualitative content analysis is suited to study different realities, descriptions and experiences of patients within a population, it was deemed as a suitable method for the present study. One crucial aspect of conducting qualitative research concerns data saturation. In our study, we included 15 patients, and we repeatedly read the interview transcripts in order to ensure credibility in the main, sub-categories, codes and condensed meaningful units, to ensure that all data was relevant for the aim of the study. During this process of the analysis, we noticed that neither further codes, nor subcategories could be extracted in the last analysed interviews. We therefore feel positive that data saturation was reached. In addition, two experts in sport and exercise psychology, highly trained in qualitative research helped in triangulating the steps of the analysis process.

### Limitations

The patients in the present study were recruited from a geographical area where a rehabilitation specific registry for ACL injury is available, which provides patients with the opportunity for continuous evaluation of progress in the rehabilitation. It is possible that the patients included in the present study might have a greater motivation towards rehabilitation than other patients, since they participate in the rehabilitation specific registry, and are continuously assessed with test of muscle function and PROs, which previously has been associated with stronger motivation for rehabilitation [40]. Another limitation is that patients were recruited from the same geographical area, therefore their experiences are influenced by the culture of the place in which they live. Furthermore, we chose to only include young active women, which is the subgroup of patients with the greatest incidence of ACL re-ruptures. A further limitation is the risk of recall bias, as several months had passed between first ACL injury and time of interview. However, the key concept in our result is that the first ACL injury is seen as a negative event, in contrast to the ACL re-rupture which had instead positive nuances. Therefore, we do not believe recall bias to have altered the loading of the feelings with regard to the ACL injury. The sharp inclusion criteria (young sports active females) limits transferability of our results, and they should therefore be appreciated with caution.

## Conclusion

Young active females experience a wide range of negative experiences going through an ACL re-rupture. The ACL re-rupture journey can end with the experience of becoming a new, stronger person. Based on the voices from patients who go on to suffer a ACL re-rupture after ACL reconstruction, there is need for better inclusion of the patients’ perspective in shared decision-making for RTS, based on the expressed mismatch between clearing patients for RTS using test batteries and whether patients feel ready to resume sport. Put into clinical perspective, these results can facilitate clinicians’ understanding of the burden of ACL re-ruptures, and hopefully encourage clinicians to further integrate patients’ perceptions in RTS criteria.

## Data Availability

The datasets generated and analysed during the current study are not publicly available due decision and recommendations made by the Swedish Ethical Review Authority. However, parts of the datasets generated and analysed during the current study are available from the corresponding author, Ramana Piussi (ramana.piussi@gu.se) on reasonable request.

## Abbreviations

ACL: Anterior Cruciate Ligament
PROs: Patient Reported Outcomes
RTS: Return To Sport
AIMS: Athletic Identity Measurement Scale.
HADS: Hospital Anxiety and Depression Scale.
MADRS: Montgomery Åsberg Depression Rating Scale.
SF-36: Short Form Health Survey

## References

[1] Frobell RB, Roos EM, Roos HP, Ranstam J, Lohmander LS (2010). A randomized trial of treatment for acute anterior cruciate ligament tears. N Engl J Med.

[2] DiSanti J, Lisee C, Erickson K, Bell D, Shingles M, Kuenze C (2018). Perceptions of rehabilitation and return to sport among high school athletes with anterior cruciate ligament reconstruction: a qualitative research study. J Orthop Sports Phys Ther.

[3] Scott SM, Perry MA, Sole G. “Not always a straight path”: patients’ perspectives following anterior cruciate ligament rupture and reconstruction. Disabil Rehabil. 2018;40(19):2311–7. 10.1080/09638288.2017.1335803 28597696

[4] Ross CA, Clifford A, Louw QA (2017). Factors informing fear of reinjury after anterior cruciate ligament reconstruction. Physiother Theory Pract.

[5] Tjong VK, Murnaghan ML, Nyhof-Young JM, Ogilvie-Harris DJ (2014). A qualitative investigation of the decision to return to sport after anterior cruciate ligament reconstruction: to play or not to play. Am J Sports Med.

[6] Nordahl B, Sjostrom R, Westin M, Werner S, Alricsson M (2014). Experiences of returning to elite alpine skiing after ACL injury and ACL reconstruction. Int J Adolesc Med Health.

[7] Wiese-bjornstal DM, Smith AM, Shaffer SM, Morrey MA (1998). An integrated model of response to sport injury: Psychological and sociological dynamics. J Appl Sport Psychol.

[8] McMurray NS, Bates NA, Fischer S, Schilaty ND, Hewett TE (2020). Investigation of primary and second anterior cruciate ligament tears using a geographic database. Int J Sports Phys Ther.

[9] Maletis GB, Inacio MC, Funahashi TT (2015). Risk factors associated with revision and contralateral anterior cruciate ligament reconstructions in the Kaiser Permanente ACLR registry. Am J Sports Med.

[10] Kaeding CC, Pedroza AD, Reinke EK, Huston LJ, Spindler KP (2015). Risk factors and predictors of subsequent ACL injury in either knee after ACL reconstruction: prospective analysis of 2488 primary ACL reconstructions from the moon cohort. Am J Sports Med.

[11] Schilaty ND, Nagelli C, Bates NA, Sanders TL, Krych AJ, Stuart MJ (2017). Incidence of second anterior cruciate ligament tears and identification of associated risk factors from 2001 to 2010 using a geographic database. Orthop J Sports Med.

[12] Beischer S, Gustavsson L, Senorski EH, Karlsson J, Thomeé C, Samuelsson K (2020). Young athletes who return to sport before 9 months after anterior cruciate ligament reconstruction have a rate of new injury 7 times that of those who delay return. J Orthop Sports Phys Ther.

[13] Grassi A, Ardern CL, Marcheggiani Muccioli GM, Neri MP, Marcacci M, Zaffagnini S (2016). Does revision ACL reconstruction measure up to primary surgery? a meta-analysis comparing patient-reported and clinician-reported outcomes, and radiographic results. Br J Sports Med.

[14] Wright RW, Gill CS, Chen L, Brophy RH, Matava MJ, Smith MV (2012). Outcome of revision anterior cruciate ligament reconstruction: a systematic review. J Bone Joint Surg Am.

[15] Kvist J, Ek A, Sporrstedt K, Good L (2005). Fear of re-injury: a hindrance for returning to sports after anterior cruciate ligament reconstruction. Knee Surg Sports Traumatol Arthrosc.

[16] Svantesson E, Hamrin Senorski E, Kristiansson F, Alentorn-Geli E, Westin O, Samuelsson K (2020). Comparison of concomitant injuries and patient-reported outcome in patients that have undergone both primary and revision ACL reconstruction-a national registry study. J Orthop Surg Res.

[17] Graneheim UH, Lundman B (2004). Qualitative content analysis in nursing research: concepts, procedures and measures to achieve trustworthiness. Nurse Educ Today.

[18] Willig C, Rogers WS. The SAGE handbook of qualitative research in psychology. London: Sage; 2017.

[19] Poucher ZA, Tamminen KA, Caron JG, Sweet SN (2020). Thinking through and designing qualitative research studies: a focused mapping review of 30 years of qualitative research in sport psychology. Int Rev Sport Exerc Psychol.

[20] Tong A, Sainsbury P, Craig J (2007). Consolidated criteria for reporting qualitative research (COREQ): a 32-item checklist for interviews and focus groups. Int J Qual Health Care.

[21] Piussi R, Berghdal T, Sundemo D, Grassi A, Zaffagnini S, Sansone M, et al. Self-reported symptoms of depression and anxiety after acl injury: a systematic review. Orthop J Sports Med. 2022;10(1):23259671211066492. 10.1177/23259671211066493 PMC8777351 35071657

[22] Ware JE Jr,  Sherbourne CD (1992). The MOS 36-item short-form health survey (SF-36). I. Conceptual framework and item selection. Med Care.

[23] Ware JEJ (2000). SF-36 health survey update. Spine.

[24] Snaith RP (2003). The Hospital Anxiety And Depression Scale. Health Qual Life Outcomes.

[25] Bjelland I, Dahl AA, Haug TT, Neckelmann D (2002). the validity of the hospital anxiety and depression scale: an updated literature review. J Psychosom Res.

[26] Montgomery SA, Asberg M (1979). A new depression scale designed to be sensitive to change. Br J Psychiatry.

[27] Brewer BW, Van Raalte JL, Linder DE. Athletic identity: Hercules’ muscles or Achilles heel? Int J Sport Psychol. 1993;24(2):237–54.

[28] Smith B, McGannon KR (2018). Developing rigor in qualitative research: problems and opportunities within sport and exercise psychology. Int Rev Sport Exerc Psychol.

[29] Graneheim UH, Lindgren BM, Lundman B (2017). Methodological challenges in qualitative content analysis: a discussion paper. Nurse Educ Today.

[30] Filbay SR, Culvenor AG, Ackerman IN, Russell TG, Crossley KM (2015). Quality of life in anterior cruciate ligament-deficient individuals: a systematic review and meta-analysis. Br J Sports Med.

[31] Filbay SR, Ackerman IN, Russell TG, Macri EM, Crossley KM (2014). Health-related quality of life after anterior cruciate ligament reconstruction: a systematic review. Am J Sports Med.

[32] Filbay SR, Ackerman IN, Russell TG, Crossley KM (2017). Return to sport matters-longer-term quality of life after ACL reconstruction in people with knee difficulties. Scand J Med Sci Sports.

[33] van Melick N, Pronk Y, Nijhuis-van der Sanden M, Rutten S, van Tienen T, Hoogeboom T. Meeting movement quantity or quality return to sport criteria is associated with reduced second ACL injury rate. J Orthop Res. 2022;40(1):117–28. 10.1002/jor.25017 33650704

[34] Welling W, Benjaminse A, Lemmink K, Gokeler A (2020). Passing return to sports tests after ACL reconstruction is associated with greater likelihood for return to sport but fail to identify second injury risk. Knee.

[35] Grindem H, Engebretsen L, Axe M, Snyder-Mackler L, Risberg MA (2020). Activity and functional readiness, not age, are the critical factors for second anterior cruciate ligament injury - the Delaware-Oslo ACL cohort study. Br J Sports Med.

[36] Arvinen-Barrow M, Clement D. The psychology of sport and performance injury: an interprofessional case-based approach. London: Routledge; 2019.

[37] Burland JP, Howard JS, Lepley AS, DiStefano LJ, Lepley LK, Frechette L (2020). What are our patients really telling us? psychological constructs associated with patient-reported outcomes after anterior cruciate ligament reconstruction. J Athl Train.

[38] Johnson U, Ivarsson A, Karlsson J, Hägglund M, Waldén M, Börjesson M (2016). Rehabilitation after first-time anterior cruciate ligament injury and reconstruction in female football players: a study of resilience factors. BMC Sports Sci Med Rehabil.

[39] Heijne A, Silbernagel KG, Lundberg M. I don't opt out of things because I think I will get a sore knee, but I don't expose myself to stupid risks either: patients’ experiences of a second ACL injury—an interview study. Knee Surg Sports Traumatol Arthrosc. 2022;30:2244–50. 10.1007/s00167-021-06762-x PMC9206613 34661692

[40] Filbay SR, Grindem H (2019). Evidence-based recommendations for the management of anterior cruciate ligament (ACL) rupture. Best Pract Res Clin Rheumatol.

